# Preclinical Experimental Study on New Cervical Implant Design to Improve Peri-Implant Tissue Healing

**DOI:** 10.3390/bioengineering11111155

**Published:** 2024-11-16

**Authors:** Sergio Alexandre Gehrke, Guillermo Castro Cortellari, Jaime Aramburú Júnior, Tiago Luis Eilers Treichel, Marco Aurelio Bianchini, Antonio Scarano, Piedad N. De Aza

**Affiliations:** 1 Department of Bioengineering, Universidad Miguel Hernandez de Elche, 03202 Alicante, Spain; piedad@umh.es; 2 Department of Biotechnology, Universidad Católica de Murcia, 30107 Murcia, Spain; 3 Department of Implantology, Bioface/Postgrados en Odontología/Universidad Catolica de Murcia, Montevideo 11100, Uruguay; guile237@gmail.com (G.C.C.); jaimearamburujunior@gmail.com (J.A.J.); 4 Department of Surgery, Faculty of Medicine Veterinary, University of Rio Verde, Rio Verde 75901-970, Brazil; tiago@unirv.edu.br; 5 Post-Graduate Program in Implant Dentistry, Federal University of Santa Catarina, Florianópolis 88040-900, Brazil; 6 Department of Innovative Technologies in Medicine & Dentistry, University of Chieti-Pescara, 66013 Chieti, Italy

**Keywords:** dental implants, macrogeometry, preclinical study, histomorphometry, implant stability, new cervical implant design, peri-implant tissue healing, rabbit animal study, bone–implant contact, tissue area fraction occupancy

## Abstract

**Objectives:** In this preclinical study, we used an experimental rabbit model to investigate the effects of a new implant design that involves specific changes to the cervical portion, using a conventional implant design in the control group. **Materials and Methods:** We used 10 rabbits and 40 dental implants with two different macrogeometries. Two groups were formed (n = 20 per group): the Collo group, wherein implants with the new cervical design were used, which present a concavity (reduction in diameter) in the first 3.5 mm, the portion without surface treatment; the Control group, wherein conical implants with the conventional design were used, with surface treatment throughout the body. All implants were 4 mm in diameter and 10 mm in length. The initial implant stability quotient (ISQ) was measured immediately after the implant insertion (T1) and sample removal (T2 and T3). The animals (n = five animals/time) were euthanized at 3 weeks (T1) and 4 weeks (T2). Histological sections were prepared and the bone–implant contact (BIC%) and tissue area fraction occupancy (TAFO%) percentages were analyzed in the predetermined cervical area; namely, the first 4 mm from the implant platform. **Results:** The ISQ values showed no statistical differences at T1 and T2 (*p* = 0.9458 and *p* = 0.1103, respectively) between the groups. However, at T3, higher values were found for the Collo group (*p* = 0.0475) than those found for the Control group. The Collo samples presented higher BIC% values than those of the Control group, with statistical differences of *p* = 0.0009 at 3 weeks and *p* = 0.0007 at 4 weeks. There were statistical differences in the TAFO% (new bone, medullary spaces, and the collagen matrix) between the groups at each evaluation time (*p* < 0.001). **Conclusions:** Considering the limitations of the present preclinical study, the results demonstrate that the new implant design (the Collo group) had higher implant stability (ISQ) values in the samples after 4 weeks of implantation. Furthermore, the histomorphometric BIC% and TAFO% analyses showed that the Collo group had higher values at both measurement times than the Control group did. These findings indicate that changes made to the cervical design of the Collo group implants may benefit the maintenance of peri-implant tissue health.

## 1. Introduction

Although dental implants generally have high success rates for the replacement of missing teeth, their long-term maintenance directly depends on the health of the adjacent tissues [1,2]. The constant evolution of the implant dentistry field has provided significant advances in the development of new dental implant designs, especially with changes in their macrogeometries [3,4], which, as demonstrated in previous studies, can produce different responses in the osseointegration process [5,6]. These changes can be divided according to three areas of the implant: (1) the cervical portion, where the main objective is maintaining the stability of the peri-implant tissues, with new configurations that aim to optimize both implant osseointegration and esthetic and functional integration, challenging conventional standards [7]; (2) the implant body, for which the most frequent modifications involve new coil designs; and (3) the apical portion, the modifications of which are related to the cutting power and threading of the implant.

The stability and maintenance of the peri-implant tissue health around the cervical portion (neck) of the implant represent the primary objectives in the contemporary practice of implant dentistry [8]. The integrity of the soft and bone tissues in this region not only directly influences the esthetics and functional comfort of patients but is also crucial for the longevity and success of dental implant treatments [8]. In this sense, the phenotype of peri-implant tissues is crucial for obtaining adequate long-term results [9,10]; however, other authors have recently associated the presence of peri-implantitis with a soft-tissue phenotype [11]. The cervical implant portion challenges professionals in the field to constantly seek new approaches and technologies that promote robust osseointegration and the healthy adaptation of soft tissues [1]. The evolution of implant designs has been guided by studies that highlight the importance of characteristics such as the shape, surface texture, and biomechanical adjustments that minimize the impact on the surrounding tissues [12].

Several studies have addressed cervical implant design and its relationship with marginal bone loss, but the results are varied in the literature [13,14,15]. Implants that present a reduction in the cervical portion (reverse concave neck) increase the bone mass in this area and, consequently, the peri-implant tissue volume [16], while implants with micro-threads in the neck can reduce the marginal bone loss compared to those with open-thread collars [17,18]. Furthermore, the reduction in the cervical portion of the implant can considerably increase the peri-implant tissue phenotype, thereby benefiting the maintenance of tissue stability in the long term and reducing the possibility of diseases in this region [11].

**Figure 1:**
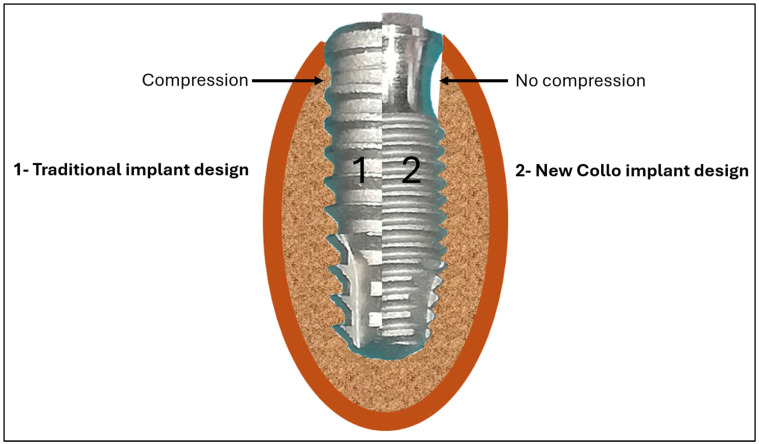
Schematic image showing the difference in the cervical portion macrogeometries between (1) a traditional implant model and (2) the new Collo implant.

A new cervical macrogeometry design was recently developed as a promising alternative to the traditional models to facilitate the formation and maintenance of peri-implant tissues through carefully designed modifications, such as the introduction of a concave area in the cervical portion, and to promote more effective osseointegration and the optimization of the biological response [19]. This new design generates a clot chamber in the cervical portion of the implant without causing compression in this area, compared to conventional implant designs (Figure 1).

However, when new changes in implantable material designs are developed, preclinical trials in animals are essential to determine whether these changes are beneficial or not [20,21]. The aim of these studies, in addition to enabling comparisons of the new proposed designs with the existing implant designs, is to determine and quantify the degree of improvement achieved. Histological images and histomorphometric analyses are important tools for this type of evaluation of new materials [22,23]. Furthermore, as the initial implant stability is essential for achieving implant osseointegration, the post-healing stability of the tissues around the implants is critical for maintaining long-term osseointegration. In this sense, new technologies—such as the Osstell device—are of considerable assistance in obtaining biomechanical data from implants [22,24].

Therefore, in this preclinical study, we used an experimental rabbit model to investigate the biomechanical and histomorphological behavior of a new implant design that involves specific changes to the cervical portion, using a conventional implant design in the control group. The following parameters were evaluated at two different times (3 and 4 weeks after the implantations): (i) the stability of the implants at different times; (ii) the bone response in the cervical area; and (iii) the characteristics of the newly formed tissue in the initial healing stages.

## 2. Materials and Methods

### 2.1. Implants and Group Formation

**Figure 2:**
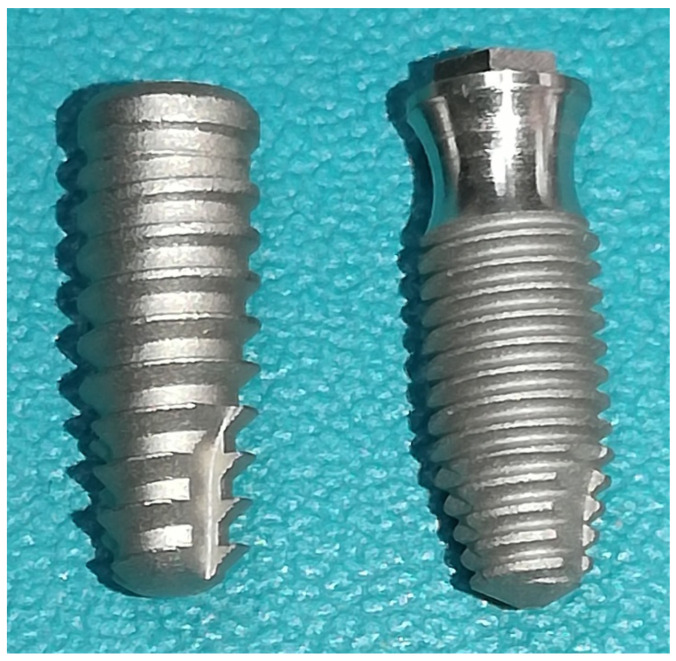
Representative images of the implant models used in this study.

A total of 40 implants were used in this study, which were divided into two groups (n = 20 per group): the Collo group, wherein implants with the new cervical design were used, which present a concavity (reduction in diameter) in the first 3.5 mm, the portion of the implant without surface treatment; the Control group, wherein conical implants with the conventional design were used, with surface treatment throughout the body. Both implants were manufactured by the company Implacil De Bortoli (São Paulo, Brazil) and were 4 mm in diameter and 10 mm in length. The surface treatment for the implants of both groups was blasted with titanium oxide and subsequent acid etching, according to information provided by the manufacturer. Figure 2 presents representative images of the implant models.

### 2.2. Animal Model and Care

In the present study, 10 adult female New Zealand white rabbits weighing between 4 and 5 kg were used. Initially, the study protocol was analyzed and approved by the Animal Experimentation Committee of the University of Rio Verde (UnRV, Rio Verde, Brazil) under number 04/2020. This animal model (rabbit) is a testing system that is frequently used for preclinical materials tests, as it presents an adequate acquisition cost, ease of handling and care, availability, tolerance to captivity, and convenience of housing [21]. Both tibias were selected as the site for installing the implants due to the simplicity of the surgical access and their anatomical characteristics (proportions of cortical and medullary bone). International guidelines for animal studies were followed, with the animals receiving the recommended care and handling protocol [25], as well as other relevant guidelines and regulations and the ARRIVE guidelines. All of the animals were kept in individual cages, with 12 h of light and food and water ad libitum throughout the experiment.

### 2.3. Implant Surgery and Distribution and Animal Euthanasia

**Figure 3:**
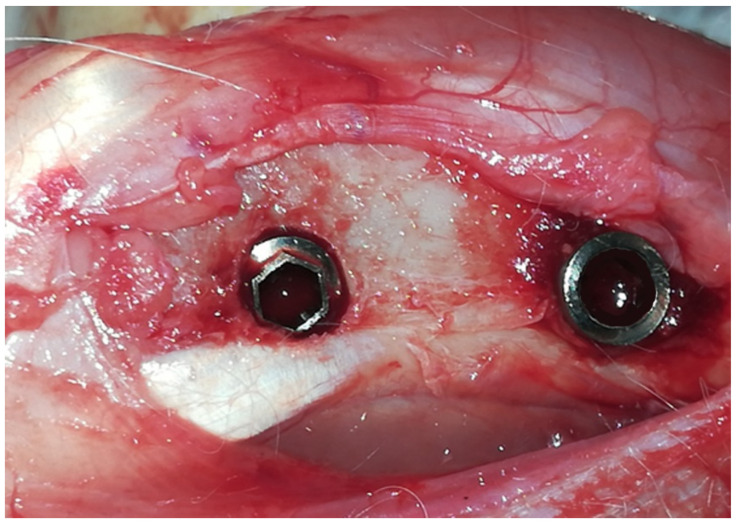
Representative image of the two implants inserted in the tibia: one at a proximal site (approximately 1 cm away from the joint) and another approximately 1 cm distally.

Two implants were installed per tibia in each animal: one at a proximal site (approximately 1 cm away from the joint) and another approximately 1 cm distally, as shown schematically in Figure 3.

These implant installation positions were determined to reduce possible differences in the anatomical characteristics of the bone (amounts of medullary and cortical bone), as the tibia has a greater amount of medullary bone and less cortical bone in this area [4]. The distribution was determined by using free software (available at www.randomizer.org, Geoffrey C. Urbaniak y Scott Plous, Middletown, CT, USA).

All animals were anesthetized with an intramuscular injection of 0.35 mg/kg of ketamine (Ketamina Agener^®^; Agener União Ltd., São Paulo, Brazil) and 0.5 mg/kg of xylazine (Rompum^®^ Bayer S.A., São Paulo, Brazil). Initially, a total incision was made from the proximal joint towards the distal of approximately 4 cm. Afterwards, the bone tissue was exposed, and drilling was carried out using the drill sequence and speed recommended by the manufacturer for each implant design under intense irrigation with a physiological solution. The implants were installed manually using a surgical torque wrench with an approximate torque of 15 Ncm. Finally, the tissues were sutured with a single stitch by using a nylon suture (Ethicon 3-0, Johnson & Johnson Medical, New Brunswick, NJ, USA). To control postoperative infection, a single intramuscular dose of 0.1 mL/kg of Benzetacil (Bayer, São Paulo, Brazil) was injected. To control pain and inflammation, three doses (one per day) of 3 mg/kg of ketoprofen (Ketoflex, Mundo Animal, São Paulo, Brazil) were administered. After 3 and 4 weeks, the animals were sacrificed via an overdose with an intramuscular injection of 105 mg/kg of ketamine (Ketamina Agener^®^; Agener União Ltd., São Paulo, Brazil) and 15 mg/kg of xylazine (Rompum^®^ Bayer S.A., São Paulo, Brazil) as anesthetics (n = 5 animals per time). Then, both tibias of each animal were removed and duly identified, and these samples were immediately immersed in a 4% formaldehyde solution, where they remained for 7 days before the treatment sequence for their inclusion in resin.

### 2.4. Implant Stability Quotient (ISQ) Measurement

Immediately after each implant installation, a magnetic sensor (SmartPeg, Gothenburg, Sweden) was threaded and manually tightened to measure the initial stability: sensor type 49 for the Control group and sensor type 1 for the Collo group. These measurements were carried out using the Osstell device (Osstell AB, Gothenburg, Sweden), which indicates a value from 0 to 100 that is used to determine the implant stability quotient (ISQ). For each sample, measurements were taken in two directions (proximodistal and anteroposterior), generating an average between these values. The ISQ was measured three times for each sample: immediately after installing the implants (Time 1) and removing the samples (Times 2 and 3).

### 2.5. Histological Preparation and Measurements 

As described in Section 2.3, samples were collected between 3 and 4 weeks after the implant placement (n = 5 animals per time). After 7 days of fixing the samples in a formaldehyde solution, they were subjected to a sequence of alcohol in progressive concentrations (50–100%) for dehydration for a period of 72 h at each concentration. Then, all of the samples were embedded in the historesin Technovit 7200 VLC (Kultzer & Co., Wehrhein, Germany) and polymerized, and these historesin blocks containing the samples (bone and implants) were cut through the central portion of each implant in a metallographic machine (Isomet 1000; Buehler, Germany), subsequently fixed on the blades, and sanded on a bench polisher (Polipan- U, Panambra Zwick, São Paulo, Brazil) using an abrasive paper sequence (180, 320, 600, 1000, and 1200 mesh). We rehydrated the slides using a decreasing sequence of alcohol (100–50%) plus a 100% ethanol solution and 10 vol hydrogen peroxide in a 50:50 ratio, and then stained them with Picrosirius red–hematoxylin. Image series were acquired by using light optical microscopy (Nykon E200, Tokyo, Japan).

**Figure 4:**
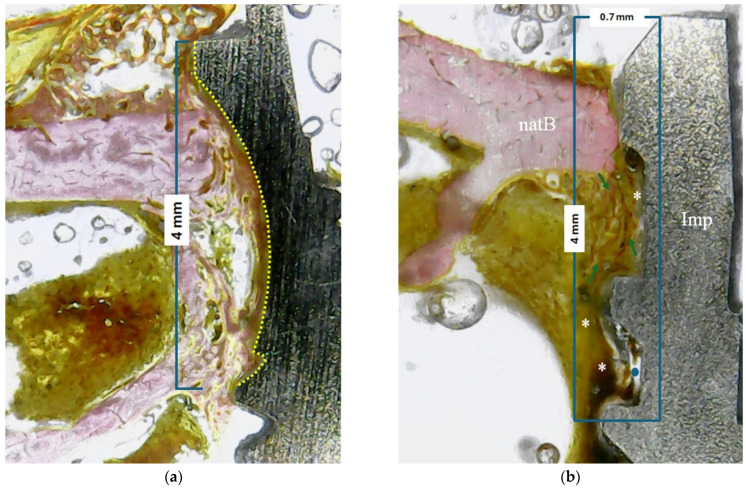
Representative images of the performed measurements: (**a**) BIC% within 4 mm from the implant platform in the apical direction (yellow line); (**b**) peri-implant tissue occupancy in a rectangular 4 × 0.7 mm area (inside the blue rectangle) (natB: native bone; 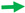: new bone; * collagen matrix; 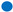: medullary space).

In all of the samples, the bone–implant contact percentage (BIC%) parameters were evaluated within 4 mm from the implant platform in the apical direction, as shown schematically in Figure 4a. Furthermore, in an area of 4 mm (from the implant platform towards the apical direction) by 0.7 mm (from the implant platform towards the native bone), the tissue area fraction occupancy percentage (TAFO%) with the anatomical characteristics of the newly formed tissue were evaluated (i.e., the amount of newly formed bone, the collagen matrix, and the medullary spaces present) (Figure 4b). ImageJ software (National Institute of Health, Bethesda, MD, USA) was used for both evaluations and measurements.

### 2.6. Statistical Analysis

The sample size was determined to achieve a power level of 85% and a significance level of 0.05 by using SigmaStat 4.0 software (Systat Software Inc., Chicago, IL, USA). Based on the desired power and the differences in the means and standard deviations between the groups, the minimum required sample size for each time/group was 8 specimens. However, 10 specimens (implants) were analyzed at each time/group.

GraphPad Prism 5.01 software (GraphPad Software Inc., San Diego, CA, USA) was used to perform the statistical analysis for all of the proposed cases, with *p* > 0.05 indicating a statistical difference. Initially, the Kolmogorov–Smirnov test was used to evaluate and confirm the normality. For the implant stability quotient analysis, intra- and intergroup one-way ANOVA tests were performed to identify possible differences. The intra- and intergroup bone–implant contact (BIC%) and tissue area fraction occupancy (TAFO%) percentages were analyzed by using the Bonferroni multiple-comparison test.

## 3. Results

**Figure 5:**
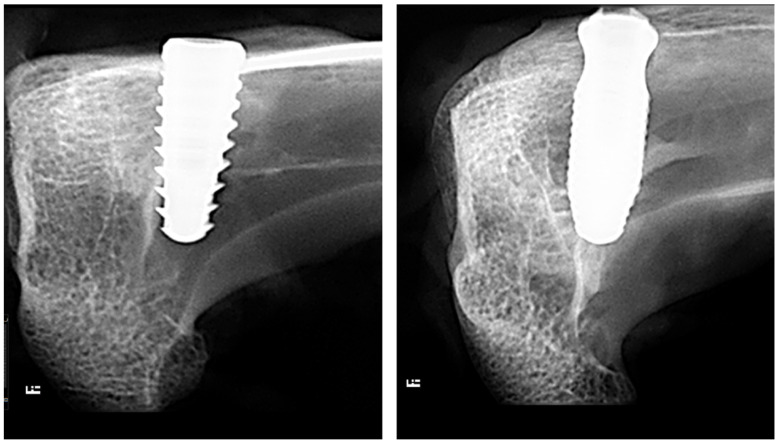
Radiographic images taken after sample removal to assess the condition of the bone tissue around the implants: Control group ((**left**) image) and Collo group ((**right**) image).

No adverse events were observed during the healing period, such as inflammation and/or infection in the surgery area. All of the implants showed clinical stability in the percussion test and radiographic signs of osseointegration during the predetermined evaluation periods (3 and 4 weeks), as demonstrated in the radiographic images shown in Figure 5.

### 3.1. Implant Stability Quotient (ISQ) Results

**Figure 6:**
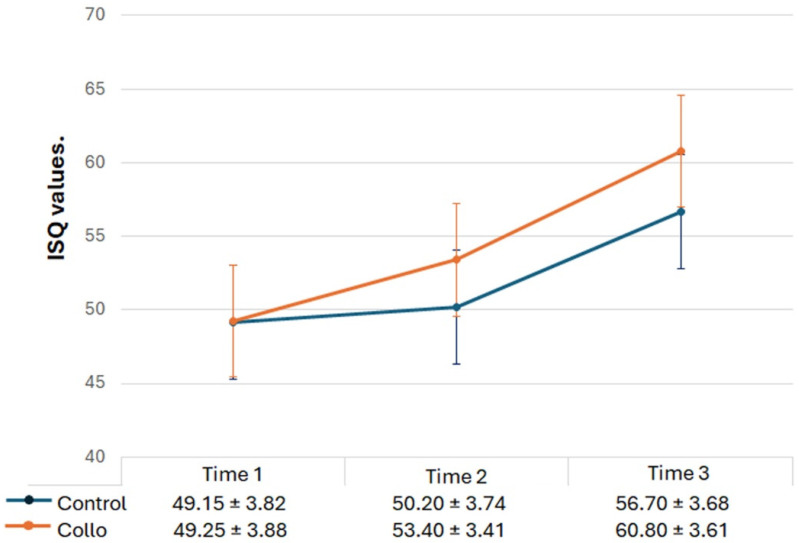
Line graph showing the ISQ evolution for both groups at the three predetermined evaluation times: Time 1: immediately after the implant installation; Time 2: 3 weeks after the implant installation; and Time 3: 4 weeks after the implant installation. Below are the averages and standard deviations of the values obtained for each group and time.

A comparison of the implant stability measured by using the Osstell^®^ (Osstell AB, Gothenburg, Sweden) device during the three evaluation periods is shown in Figure 6. Statistically, there were differences between the three evaluation times in both groups: intragroup (ANOVA): *p* = 0.0003 for the Control group, and *p* = < 0.0001 for the Collo group; intergroup (*t*-test): no statical differences were detected between the groups at Time 1 (*p* = 0.9458) and Time 2 (*p* = 0.1103). However, statistical differences were detected between the groups at Time 3 (*p* = 0.0475). 

### 3.2. Histological Results

**Figure 7:**
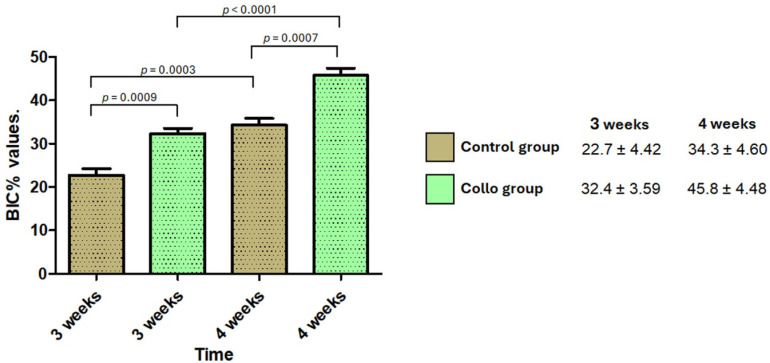
Bar graph showing the BIC% values and statistical differences between the groups. On the right are the means and standard deviations of both groups and times.

Samples from the Collo group presented higher BIC% values than those of the Control group at Time 2 (3 weeks) and Time 3 (4 weeks). The data obtained (means and standard deviations) and a statistical comparison are presented in Figure 7.

**Figure 8:**
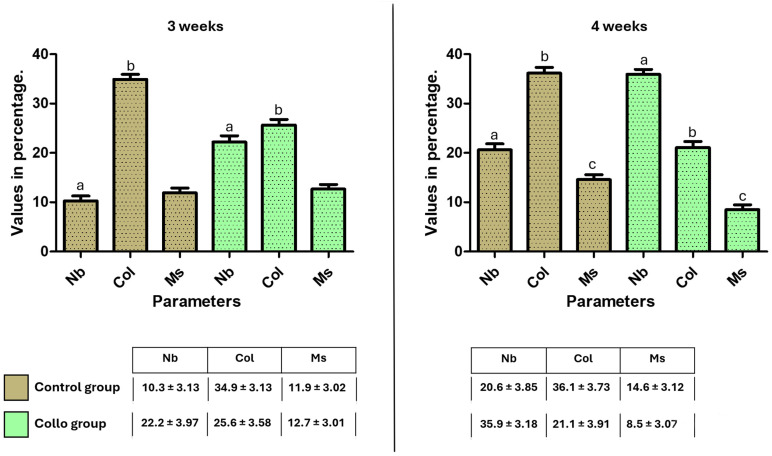
Bar graph showing the TAFO% values and statistical differences between the groups. a, b, and c show the statistical differences in the same parameters between the groups; in all cases, *p* < 0.001. Nb: new bone presence; Col: collagen matrix; Ms: medullary spaces. Below are the averages and standard deviations of the values obtained for each group and time.

Figure 8 presents the means and standard deviations of the TAFO% values of the rectangle area in the cervical position, where the predetermined parameters were measured (the new bone, collagen matrix, and medullary spaces). Furthermore, the statistical differences in the same parameters at each evaluation time between the groups are shown in the bar graph figure.

## 4. Discussion

In this study, we performed a comparative analysis between a new implant design with changes in the cervical portion (the Collo group) and a conventional implant (the Control group), focusing on the initial osseointegration stages in a preclinical trial. The main parameters evaluated were the initial stability of the implants (measured by ISQ) at the time of installation and the stability at 3 and 4 weeks after installation. Additionally, histological analyses were performed to evaluate the bone–implant contact (BIC%) and tissue area fraction occupancy (TAFO%) proportions of the predetermined cervical portion (the initial 4 mm from the platform). According to the results, the Collo group presented higher stability values (ISQ), both at the time of installation and in the subsequent measurements after 3 and 4 weeks, than those of the Control group. Furthermore, according to the histological evaluations, the Collo group had a higher proportion of bone–implant contact (BIC%) and a higher rate of new bone formation (TAFO%) at both evaluation times. These findings are consistent with previous studies that suggested that macrogeometric changes in implants can significantly influence the osseointegration process and implant stability quotient values at different times [5,26]. Furthermore, changes in the macrogeometry and shapes of the external threads of dental implants are still topics that are widely explored in the literature [27,28,29,30].

Several studies on macrogeometric changes have been conducted that focus on animal models, with most of them exploring the biological mechanisms involved in the healing process with different surface treatments and macrogeometries [31,32,33,34]. Implant macrogeometry is one of the factors that has the greatest influence on primary implant stability and initial torque; consequently, it promotes osseointegration optimization [35]. The positive results for the tissue neoformation around the cervical portion of the Collo implants compared with those of the Control group corroborate the results of other studies that show that the proposed change in the implant macrogeometry can have a significant impact on the osseointegration process [5,26,35]. Specifically, the more accelerated formation of peri-implant tissues is highlighted, which is attributed to the presence of a bone non-compression area due to the formation of a healing chamber, as shown in Figure 1. Furthermore, previous studies have shown that the presence of clot chambers in dental implants can optimize osseointegration [5,26,31,36] and promote an environment that is conducive to cell differentiation and tissue neoformation around the implant, which are essential for successful long-term osseointegration. Other studies have shown that, in conjunction with appropriate surface treatments and an adequate macrogeometry, healing chambers can accelerate healing and promote more effective osseointegration [36,37].

The bone portion in contact with the cervical area of the implant is characterized by the presence of cortical bone, which is directly related to the initial implant stability [38,39]. However, the cortical bone tissue presents anatomical characteristics with little vascularization compared to the medullary bone portion. Therefore, the compression of this area (cortical) can cause unwanted effects, such as ischemia, necrosis, and bone resorption [40,41]. Other authors indicated that the highest strain during implant insertion, along with the greatest compressive force and shear stresses, occurs in the cortical bone portion, particularly in the region of the first implant thread [42]. Thus, in the new Collo implant macrogeometry, a compression-free area is formed on the cortical bone tissue, which appears to be effective for healing events based on the results obtained. Other authors have recently proposed the incorporation of cutting blades into the implant’s cervical portion with the same purpose of reducing compression on the cortical bone during implant installation [43,44]. The same animal model (rabbits) and area (tibia) were used in these studies as in this study, and the results show that this strategy is adequate and can prevent marginal bone loss around implants, corroborating the results found in the present study. Other changes in cervical implant designs have been proposed and tested, all emphasizing the non-compression of the cortical bone, resulting in less marginal bone loss [45,46,47].

The initial stability values measured using resonance frequency analysis with the Osstell device, which is an effective and non-invasive method [48], were similar for both groups, with no statistical differences between them. These findings demonstrate that the presence of the healing chamber, provided via the cervical design of the Collo implants, did not reduce the initial ISQ values, as the final part of the implant platform (0.5 mm) was anchored in the cortical bone. In this sense, as demonstrated by other authors, contact between the cervical portion of the implant and the cortical bone favors initial stability [39]. At the other predetermined measurement times (3 and 4 weeks after the implantations), the values were higher for the Collo group than those for the Control group, corroborating findings from other studies that relate changes in macrogeometry to the ISQ values obtained [5,26].

The macrogeometry is also capable of influencing the biomechanics and distribution of chewing loads [49]. However, studies have shown that the primary stability may not be directly related to the insertion torque [50]. Therefore, some authors question the correlation between high insertion torque and high clinical success rates for dental implants [51], as it can result in high tension in the peri-implant region and, consequently, provoke negative responses in both the biological and secondary stabilities. The macrogeometry stands out as one of the factors that can directly interfere in dental implant osseointegration, enabling a reduction in the loading time [5,26,52].

Finally, this study was a preclinical trial, and the results were observed under controlled conditions and may vary in real clinical settings, where additional factors such as individual patient characteristics, oral health conditions, and implantation techniques may influence the results. Subsequent clinical studies are needed to confirm the applicability of these findings to everyday dental practice and to evaluate the long-term effectiveness of this new implant design.

One of the limitations of this preclinical study is that it was conducted on small animals (rabbits) and in an area free of contamination (tibia), which is completely different from the oral cavity, where there are many variables that can alter the results. Despite this, this experimental model is widely used and is considered ideal for the evaluation of the tissue response around implants (osseointegration) [21].

## 5. Conclusions

According to the results of this preclinical study, the new Collo implant design showed superior implant stability (ISQ) values in the samples after 4 weeks of implantation. Furthermore, the histomorphometric analysis of the BIC% and TAFO% showed that the Collo group had higher values at both measurement times than those of the Control group. These findings show that changes made to the cervical design of the Collo implants may benefit the maintenance of peri-implant tissue health, mainly due to the increase in the tissue volume in this area. Subsequent clinical studies are needed to validate these findings and verify their large-scale clinical applicability.

## Data Availability

All relevant data are contained within the paper.
